# Severe Keratitis Caused by *Pseudomonas aeruginosa* Successfully Treated with Ceftazidime Associated with Acetazolamide

**DOI:** 10.1155/2009/794935

**Published:** 2009-04-26

**Authors:** Benoit Hue, Marc Doat, Gilles Renard, Marie-Laure Brandely, François Chast

**Affiliations:** ^1^Department of Pharmacy, Pharmacology, and Toxicology, Hotel-Dieu, 1 Place Notre-Dame, 75004 Paris, France; ^2^Department of Ophthalmology, Hotel-Dieu, 1 Place Notre-Dame, 75004 Paris, France

## Abstract

*Purpose*. To report a case of microbial keratitis caused by Pseudomonas aeruginosa treated with a combination of acetazolamide and ceftazidime. *Methods*. Case report. *Results*. We report the case of a 17-year-old contact lens-wearing female who developed severe keratitis due to *Pseudomonas aeruginosa* temporarily healed with topical fortified antibiotic eye drops. After few days, the patient relapsed, and topical and intravenous ceftazidime were added. Concomitantly, oral administration of acetazolamide was prescribed. This carbonic anhydrase inhibitor was added to the antibiotic regimen in order to decrease the anterior chamber pH, and then, the ceftazidime ionization. By lowering the state of ionization of the antibiotic in the aqueous humor, its concentration was increased. This was confirmed by an improvement of the patient within few days and a rapid eradication of the infection. *Conclusion*. This is the first reported case of keratitis caused by *P. aeruginosa* successfully treated using acetazolamide as an enhancer of ceftazidime effectiveness.

## 1. Case Report

A 17-year-old woman was referred with a 48-hour history of a painful left eye as a consequence of a severe keratitis most likely of bacterial aetiology. Pain started after bathing in a public swimming pool while continuing to wear her contact lenses on a daily basis. On presentation, the left eye visual acuity was 2.3 LogMar (L). An extensive necrotic stromal infiltrate was observed in the cornea with a dense hypopyon. The corneal ulcer size was 7 mm × 7 mm. Cornea scrapings were performed for microbiological staining and inoculated for culture. On admission, topical treatment included vancomycin 5%, ticarcillin 0.66%, and amikacin 3% eye drops administered hourly in the eye. On day 2, the bacterium was identified as *Pseudomonas aeruginosa* and antibiotic disc sensitivity testings revealed that the organism was sensitive to most of antibiotics including ticarcillin, amikacin, ciprofloxacin, and ceftazidime. Initial treatment was changed to ciprofloxacin 0.3% (Ciloxan, Alcon, France), ticarcillin 0.66%, and amikacin 3% (prepared by the hospital pharmacy). An amoebic polymerase chain reaction (PCR) gave negative results. Six days after admission, ulcer size and hypopyon were reduced, and the patient was discharged from hospital but prescribed to continue the topical antibiotic treatment at home, every 4 hours.

**Figure 1:**
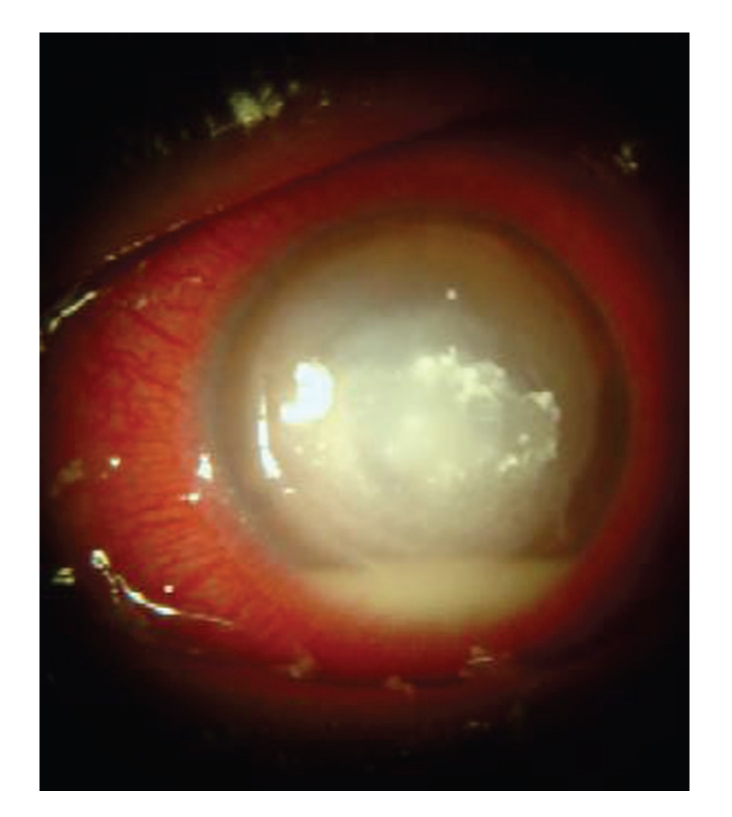
Three weeks after initial presentation, corneal ulcer associated with an intense inflammation and a dense hypopyon were still observed.

The patient represented to the emergency unit, on day 13. Ulcer size had increased and hypopyon was observed again. It was decided to hospitalize the patient again. Initial treatment with topical vancomycin, ticarcillin, and amikacin eye drops was restarted. On day 16, due to the inefficacy of this treatment, corneal scrapings were performed again for bacterial culture and amoebic PCR, results of which were both negative. Three weeks after the start of the infection, the clinical situation was still worsening and the integrity of the eye was compromised (Figure 1).

At this stage, a new therapeutic strategy was prescribed; topical and intravenous ceftazidime were included in the antimicrobial strategy. A fortified 5%-ceftazidime ophthalmic solution was prepared by the hospital pharmacy. It was instilled hourly in the infected eye. Additionally, a 1-g intravenous injection of ceftazidime (Fortum, GSK, Paris) was administered every 8 hours in association with 250-mg oral acetazolamide [1] (Diamox, Sanofi-Aventis, France) every 24 hours. After 2 days (Day 23), clinical improvement was shown according to corneal abscess regression and hypopyon disappearance.

**Figure 2:**
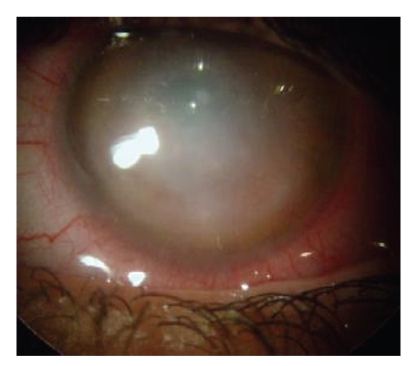
Four weeks after the start of ceftazidime treatment, we noticed a central corneal scar, absence of hypopyon, and no evidence of active keratitis.

One week after the initiation of this late treatment (Day 30), clinical improvement allowed a cessation of the intravenous ceftazidime and a reduction in the frequency of instillation of the topical fortified antibiotics. Ceftazidime was tapered gradually over the next three weeks and eventually stopped. Abscess regression and hypopyon disappearance are illustrated by Figure 2.

**Figure 3:**
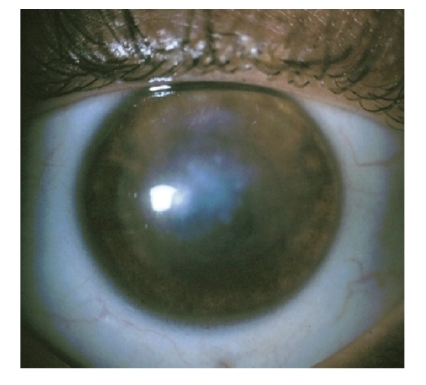
A slit lamp photograph of the left eye 4 months after initial presentation illustrates a central corneal scar without inflammation or neovessels.

Four months after the first hospitalization, the left eye visual acuity was 0.6 L. There was evidence of central corneal scarring without inflammation or neo-vessels (Figure 3).

## 2. Discussion

*P. aeruginosa* is the predominant Gram-negative bacterium that infects the cornea. For many years, keratitis caused by *Pseudomonas* has been commonly associated with the wearing of soft contact lenses [2], and it has recently been reported that the wearing of contact lenses was the most common risk factor for keratitis and that the most commonly isolated organism was *P. aeruginosa* [3, 4]. Even in compliant patients [5], standard lens care hygiene precautions are probably not sufficient for preventing the development of sporadic corneal infections.

In this case, topical broad-spectrum antibiotics were used initially in the empiric treatment [6, 7] and then switched, once the results of cultures and antibiotic sensitivity patterns were recovered from the laboratory. Two weeks after the start of treatment, scrapings were performed again but, this time, without pathogen recovery.

Three weeks after first admission to hospital, the integrity of the eye was compromised. At this stage, a topical and intravenous ceftazidime regimen was started [1, 8]. Ceftazidime is a third-generation cephalosporin antibiotic, with an extended spectrum of activity against Gram-negative bacteria, especially *Pseudomonas spp*. It is one of the most effective antibiotics against *P. aeruginosa*, the organism isolated after first corneal scrapings. Topical fortified 5%-ceftazidime eye drops (50 mg/mL) were prepared (in an appropriate compounding aseptic containment isolator maintaining an ISO 5 air quality environment) in saline, following data showing a short-term stability (4 days at 4°C) [9]. Ceftazidime pharmacokinetic properties in healthy volunteers give satisfactory penetration features of the antibiotic into aqueous humor; a mean concentration of 11 ± 4 *μ*g/mL (after a 2-g ceftazidime infusion), corresponding to a penetration ratio of 19%, has been reported [8].

This case report illustrates the fact that concentrations obtained with topical antibiotic treatment alone, even if delivered on an inflamed cornea may not be sufficient to eradicate intermediate or poorly sensitive *Pseudomonas* strains. In such cases, it is necessary to add systemic treatment to the topical antibiotic regimen. We also decided to enhance the penetration of the antibiotic in the anterior chamber by the means of an unusual but useful pharmacokinetic interaction with acetazolamide. Concomitant administration of acetazolamide was decided owing to a previous report demonstrating that it increases aqueous humor concentration of intravenous ceftazidime. It has been established that 2, 4, and 6 hours after a 2-g ceftazidime infusion, antibiotic concentration in aqueous humor was significantly increased after acetazolamide (250 mg for every 6 hours) coadministration [1].

Addition of acetazolamide, a carbonic anhydrase inhibitor (CAI), in the therapeutic regimen, allows an increase in the concentration of ceftazidime in the anterior chamber by a factor 2 to 5. For instance, 2 hours after the end of the infusion, concentrations of 4.26 ± 1.75 mg/L and 22.4 ± 16.6 mg/L were measured, respectively, in aqueous humor in patients without or with acetazolamide treatment [1]. For *P. aeruginosa*, ceftazidime (Minimal Inhibitory Concentrations) MICs range from 2 to 6 mg/L [10]. These data suggest that the use of acetazolamide, by increasing the drug concentration at its site of action 2-to 5-fold, is likely to allow exceeding MICs for most *P. aeruginosa* strains.

In the present case, the length of the treatment was one week for intravenous ceftazidime associated with acetazolamide and one month for topical ceftazidime. The choice of acetazolamide was based on its specific pharmacological properties on the electrolytic balance in body fluids. Carbonic anhydrase has the property to reversibly accelerate the reaction between carbonic anhydride (CO_2_) and water (H_2_O). The presence of this enzyme in ocular structures, including the ciliary body processes, and the high concentration of bicarbonate in the aqueous humor allow an opportunity for CAI to play an important role in ionic changes in both posterior and anterior chambers. Within the anterior chamber, besides lowering intraocular pressure, CAI will also lower the amount of bicarbonate (HCO_3_^−^) production, [11] thus decreasing the pH. As a consequence of this phenomenon, the state of ionization of numerous pharmacological agents is more or less modified. CAI will increase the ionization of basic molecules and decrease the ionization of acidic drugs. It is well known that nonionized molecules can easily diffuse across cell membranes. In contrast, the ionized molecules are usually unable to penetrate the lipid membrane because of their low lipid solubility. Ceftazidime, with two carboxylic moieties, behaves as a weak acidic molecule in biological fluids. After CAI intake, the amount of bicarbonate in aqueous humor is lowered and the fluid is acidified as a result. In such conditions, ceftazidime ionization decreases, and its intracellular penetration is then enhanced.

Further investigations and additional cases must be reviewed before definite conclusions can be made as to the improvement in ceftazidime effectiveness when administered with acetazolamide, to treat severe keratitis caused by *P. aeruginosa*.

The anterior chamber is a crucial target when considering that most ocular infections start with germ penetration through the cornea, followed by colonization of the aqueous humor. In various acute microbial keratitis, the use of acetazolamide could be relevant when the antibiotic treatment is comprised of a weak acidic molecule (carbenicillin, ticarcillin). In such cases, acetazolamide adjunction to antibiotic treatment could be an easy and inexpensive way to obtain an increase in antibiotic concentration within the anterior chamber, allowing the antibiotic MIC to be reached more easily and facilitating improvement or healing.
